# Comparative genomics and characterization of highly adhesive and stress resistant *Lacticaseibacillus paracasei* strain E

**DOI:** 10.1016/j.crfs.2025.101245

**Published:** 2025-11-15

**Authors:** Nina Zhang, Antti Karkman, Kaisa Hiippala, Reetta Satokari, Timo M. Takala, Per E.J. Saris

**Affiliations:** a Department of Microbiology, Faculty of Agriculture and Forestry, University of Helsinki, Helsinki, Finland; b Human Microbiome Research Program, Faculty of Medicine, University of Helsinki, Helsinki, Finland

**Keywords:** *Lacticaseibacillus paracasei*, Pili, Adhesion, Genome analysis, Probiotic properties

## Abstract

*Lacticaseibacillus paracasei* strains have a long history of safe use in food products, particularly fermented foods such as yogurt and cheese. These strains are known for their health-promoting probiotic attributes. Recently, we *de novo* assembled the whole genome of *Lacticaseibacillus paracasei* subsp. *paracasei* strain E, originally isolated from cheese and later studied as an adjunct starter in cheesemaking. In this study, the strain's probiotic properties and their potential genetic basis were analyzed using the combination of *in vitro* and *in silico* methods. This analysis particularly focused on correlating specific phenotypic traits with the presence or absence of specific gene sets. The strain E harbours two copies of the same *spaCBA*-*srtC1* pilus gene cluster as *L. rhamnosus* GG, and it expresses more SpaC pilins, even though the overall adhesion to mucus was found to be somewhat lower than that of LGG. The strain E exhibited better tolerance to simulated gastrointestinal condition and remarkably higher cell surface hydrophobicity than LGG, suggesting distinct surface properties and hence a different preference for binding to host cells. Taken together, these findings indicate that *L. paracasei* E has potential as a probiotic strain.

## Highlights

• *L. paracasei* E expressed more SpaC pilins than *L. rhamnosus* GG.
• *L. paracasei* E demonstrated high tolerance in simulated gastrointestinal fluid.
• *L. paracasei* E cell surface is highly hydrophobic whereas the cell surface of *L. rhamnosus* GG is hydrophilic.
• *L. paracasei* E adheres to epithelium, highlighting its potential for food applications.

## Graphical abstract

**Figure:**
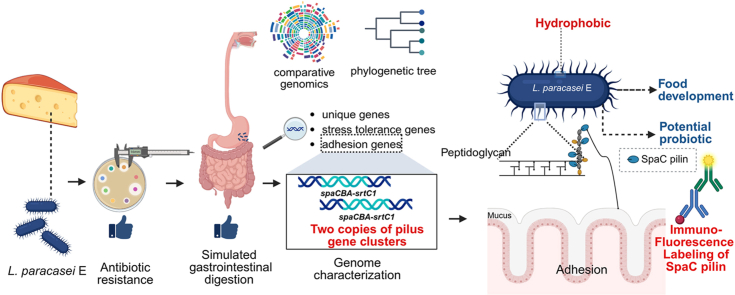

## 1 Background

*Lacticaseibacillus paracasei,* originally classified under the genus *Lactobacillus*, is now part of the newly reclassified *Lacticaseibacillus genus,* which underwent taxonomic revision in the early 2020s (Zheng et al., 2020). As of April 2025, the genus consists 36 species (Schoch et al., 2020). Before *L. paracasei* taxonomic reclassification, the nomenclature of this species underwent changes as well. Prior to 2009, most strains were designated either as *Lactobacillus casei* or *Lactobacillus paracasei* subsp. *paracasei* (Huang et al., 2018). Now, *L. paracasei* has 2 subspecies, namely *paracasei* and *tolerans*. *L. paracasei* metabolizes carbohydrates to lactic acid, thus belonging to the lactic acid bacteria (LAB) group (Raj et al., 2022). This species is commonly used as a starter culture in fermentations, plays an indispensable role in preventing food spoilage by lowering the pH, and thus can be found in a variety of fermented food products such as kefir, juice and cheese (Bettera et al., 2023; Kiousi et al., 2022). Some strains of *L. paracasei* are well known for their health-promoting probiotic properties and have obtained GRAS (Generally Recognized as Safe) status from U.S. Food and Drug Administration (FDA), such as *L. paracasei* Shirota in Yakult fermented milk product, and *L. paracasei* F-19 in yogurt (Moiseenko et al., 2023). The GRAS status was granted following extensive animal experiments and clinical trials, which demonstrated the safety and efficacy of these strains. Moreover, several other strains of *L. paracasei* have been clinically studied as well, and some have demonstrated beneficial effects on human health. For example, *L. paracasei* HA-196 has been shown to alleviate Irritable Bowel Syndrome (IBS) symptoms (Lewis et al., 2020), and *L. paracasei* HII01 supplementation significantly improved glycemia and inflammatory biomarkers in type 2 diabetes patients (Toejing et al., 2021). *L. paracasei* is proposed to exert its beneficial effects on the host by enhancing the immune system, maintaining a balanced gut microbiota, and reducing inflammation (Rastogi and Singh, 2022; West et al., 2013). These positive outcomes may be achieved through the bacterium's ability to competitively colonize the gut over pathogens and through the interaction of its metabolites with epithelial cells (Miao et al., 2023).

In this study, we aimed to characterize the probiotic potential of *L. paracasei* strain E, isolated in the 1960's from industrial high-quality cheese. In our previous studies, we developed a food-grade gene overexpression host-vector system for the strain E, and used it in cheesemaking (Navidghasemizad et al., 2013; Takala et al., 2003). The strain was found to grow and survive well in Edam cheese (Navidghasemizad et al., 2013). Taken together, these results and the proposed beneficial health effects of *L. paracasei* encouraged us to further investigate the strain E. We hypothesize that this strain possesses desirable attributes that make it a promising candidate for functional food production. Previously, we sequenced the whole genome of the strain, and subsequently, we performed genome functional annotation, comparative genomic analysis, and *in vitro* phenotypical assays to investigate its potential. Collectively, these analyses show that the strain E carries interesting and useful properties of probiotic bacteria.

## 2 Methodology

### 2.1 Genome functional annotation

Genome assembly and annotation of *L. paracasei* E were previously reported on our earlier publication (Zhang et al., 2025). Functional annotation of the genome was carried out using eggNOG-mapper (Cantalapiedra et al., 2021), BlastKOALA (Kanehisa et al., 2016) and KEGG Mapper (Kanehisa et al., 2022).

### 2.2 Identification of virulence factors, antibiotic resistance genes, prophages, and bacteriocins production

Genes involved in antibiotic resistance were investigated using RGI (Resistance Gene Identifier) v6.0.3 (Alcock et al., 2023). Genes coding for virulence factors were determined with VirulenceFinder 2.0.5 (Malberg Tetzschner et al., 2020). PHASTEST web server was used to search for prophages (Wishart et al., 2023). Bacteriocin genes were searched using the BAGEL4 database with default parameters (van Heel et al., 2018). The genome was searched for the presence of CRISPR arrays using the website server CRISPRCasFinder (Couvin et al., 2018). Only CRISPR arrays with evidence 4 were used.

Additionally, antibiotic susceptibility was assessed using the disk diffusion method (Kirby-Bauer test). Briefly, 200 μL of the overnight bacterial culture was mixed with 1 mL of phosphate-buffered saline (PBS) and uniformly spread onto the surface of an MRS agar plate. Excess liquid was removed, and the plate was allowed to dry under a laminar flow hood. Antibiotic-impregnated disks were then placed on the agar surface. After incubation at 37 °C for 18–24 h, the zones of inhibition around each disk were measured to determine the susceptibility of the bacteria to each antibiotic. The tested antibiotics and their disc contents were as follows: pristinamycin (15 μg), novobiocin (5 μg), bacitracin (10 UI), colistin (10 μg), erythromycin (15 μg), cephalothin (30 μg), tetracyclines (80 μg), streptomycin (100 μg), ciprofloxacin (10 μg), neomycin (120 μg), chloramphenicol (60 μg), ampicillin (33 μg), penicillin (5 μg), oxacillin (5 μg), gentamicin (40 μg), amoxicillin (30 μg), trimethoprim (5 μg), vancomycin(30 μg).

Antibacterial activity was also determined against selected foodborne pathogens, including *Bacillus cereus*, *Listeria monocytogenes*, and *Staphylococcus aureus,* using spot-on-lawn method. Pathogen-inoculated plates were prepared in the same manner as for the antibiotic susceptibility assay. Subsequently, 10 μL of an overnight culture of *L. paracasei* E, as well as 10 μL of its cell-free supernatant (obtained by centrifugation at 5000×*g* for 5 min and filtration through a 0.22 μm membrane), were spotted onto the surface of the pathogen-containing plates. The plates were then incubated at 37 °C for 24 h, and zones of inhibition were measured to evaluate antibacterial activity.

### 2.3 Comparative genomics

Anvi'o v 8.0 (Delmont and Eren, 2018) was used to analyse and visualize the pangenome of *L. paracasei*. Single-copy core genes were identified, concatenated, and aligned using the *anvi-get-sequences-for-gene-clusters* and *concatenate-gene-clusters* programs within anvi'o. A phylogenomic tree in NEWICK format was then generated from the concatenated gene alignment using the *anvi-gen-phylogenomic-tree* program. The resulting phylogenetic tree was visualized and annotated using the online tool Interactive Tree of Life (iTOL) v7 (Letunic and Bork, 2007). Additionally, average nucleotide identity (ANI) was calculated using the pyANI program (Pritchard et al., 2016) integrated in anvi'o.

### 2.4 Survival under gastrointestinal tract conditions

The survival test was based on previously used methodology with some changes (Ispirli and Dertli, 2017). Briefly, LGG and *L. paracasei* E cells were cultured overnight in MRS (Neogen Corporation, Lansing, USA) and collected by centrifugation at 4000×*g* for 5 min at 4 °C. Then, cells were resuspended in PBS to obtain an OD600nm of 1.0, and the number of cells (cfu/mL) was further determined by plate count method. Four mL of simulated gastric fluid (PBS, pH 3.0) with 2000 U/mL pepsin (Thermo Fisher Scientific Inc., Waltham, MA, USA) was combined with 1 mL of the bacterial suspension. The mixture was incubated for 2 h at 37 °C with agitation at 110 rpm. After incubation, the cells were harvested by centrifugation and then resuspended in 1 mL of simulated intestinal fluid with 3 % pancreatin (Sigma-Aldrich, Merck KGaA, Darmstadt, Germany) (Brodkorb et al., 2019) and 0.3 % bovine bile salts (Sigma-Aldrich) at pH 7.0 for further 2 h in the same conditions as previously. Bacterial viability was assessed by plate counts before and after the treatments.

### 2.5 Quantification of pilins using immuno-fluorescence labeling

The pilus protein SpaC on the cell surface was quantified essentially according to the method described by Rasinkangas et al. (2014). Overnight MRS cultures of *L. paracasei* E, LGG, and *L. rhamnosus* LC705 cells were collected by centrifugation at 5000×*g* for 5 min, washed with PBS for 3 times, and resuspended in PBS to OD600nm of 1.0. One mL of cell suspension was added to a 2 mL Eppendorf tubes. Ten μL of primary rabbit polyclonal antibody against LGG SpaC was added, and the mixture was incubated for 45 min at room temperature. Afterwards, cells were washed 3 times with PBS to remove unspecific bound antibodies. Cells were resuspended in 1 mL of Alexa Fluor 488-labeled goat anti-rabbit antibody (Invitrogen, Thermo Fisher Scientific Inc.) diluted 1:1000 in PBS solution and then incubated for 30 min at room temperature in dark. After incubation, the cells were washed 3 times and resuspended in 1 mL of PBS and 100 μl cell suspension was pipetted to 96-well microplate (ViewPlate, PerkinElmer Scientific). The intensity of fluorescence was measured with TECAN SPARK plate reader (Tecan Group Ltd., Männedorf, Switzerland). In addition, cell suspensions were diluted 10-fold with PBS, 100 μl aliquot was pipetted into the microplate, and the plate was placed in dark at 4 °C for overnight to let cells to settle down onto the bottom of the plate for photographing. Fluorescence image was taken by ImageXpress Nano Automated Imaging System (Molecular Devices, LLC., San Jose, CA, USA).

### 2.6 Cell surface hydrophobicity

The bacterial cell surface hydrophobicity was measured according to Jang et al. (2019), using three different solvents: hexadecane (apolar/hydrophobic), chloroform (electron acceptor/Lewis acid), and ethyl acetate (electron donor/Lewis base). The LGG and *L. paracasei* E were incubated overnight in MRS and harvested by centrifugation 5000×*g* for 5 min. The cells were washed twice with PBS and resuspended in PBS buffer with the optical density of 0.5 ± 0.02 at 600 nm (OD600nm). Three mL of the resuspended cells was mixed with 1 mL of each solvent and pre-incubated for 10 min at 37 °C. The mixture was vigorously vortexed for 1 min forming an emulsion, and incubated at 37 °C for 20 min. During incubation, the mixture separated into two phases. The OD600nm of the aqueous phase was measured.

### 2.7 *In vitro* adhesion assay

The bacterial cell adhesion of mucus was adapted from Hiippala et al. (2022). Briefly, *L. paracasei* E, LGG, and *L. rhamnosus* LC705 cells were inoculated into MRS broth supplemented with 10 μL/mL of 17 Ci/mmol [6-^3^H] thymidine (PerkinElmer Scientific, Springfield, CT, USA) and incubated at 37 °C overnight. Meanwhile, to prepare the mucus-coated plates, 100 μL of a 0.5 mg/mL mucus (Sigma-Aldrich) working solution was added to each well of microplates (Nunc, Thermo Fisher Scientific Inc.) and incubated overnight at 4 °C. Prior to use, the coated plates were washed 3 times with PBS to remove any unbound mucus. Subsequently, cells were harvested by centrifugation at 5000×*g* for 5 min, washed and resuspended in PBS, and adjusted to optical density (OD600nm) of 0.25. One hundred μL of the radiolabelled bacterial cell suspension, with or without rabbit polyclonal SpaC antiserum (Tytgat et al., 2016) were added to the precoated plates and incubated at 37 °C for 1 h. Similarly, 100 μl of OD-adjusted [^3^H]Thymidine labeled bacterial suspension in triplicate was pipetted into Optisafe HiSafe™ 3 liquid scintillation cocktail (PerkinElmer Scientific) to serve as a control. The wells containing mucus and samples were washed 3 times with PBS to remove non-adherent cells and treated with 1 % SDS −0.1 M NaOH at 37 °C overnight to lyse adherent cells. After pipetting cell lysates into liquid scintillation cocktail, the radioactivity of lysates and corresponding controls were measured with the Wallac Winspectral 1414 liquid scintillation counter (PerkinElmer Scientific). The percentage of adhered bacteria was calculated by comparing the scintillation (CPM) of samples to their corresponding controls.

### 2.8 Statistical analysis

Statistical analysis was performed using SPSS v27 and R v2025.05. Prior to interpreting the ANOVA results, the Q-Q plot evaluated the normality of the residual data distribution, and Levene's test determined the homogeneity of variances. Appropriate statistical methods were applied according to the specific experimental design, as indicated in the corresponding figure annotation.

## 3 Results & discussion

### 3.1 Genome features and functional annotation

In our previous study, we sequenced the genome of *L. paracasei* strain E and found that it consists of a 2.98 Mb chromosome and 8 plasmids of sizes between 4 and 78 kb (Zhang et al., 2025). The strain is classified as a nomadic *Lacticaseibacillus*. Nomadic lactobacilli are characterized by their larger genome size compared to host-specific strains (Kiousi et al., 2022). An increased genome size suggests enhanced adaptability to diverse environments such as plants, dairy products, and the human gut (Kiousi et al., 2022).

**Fig. 1:**
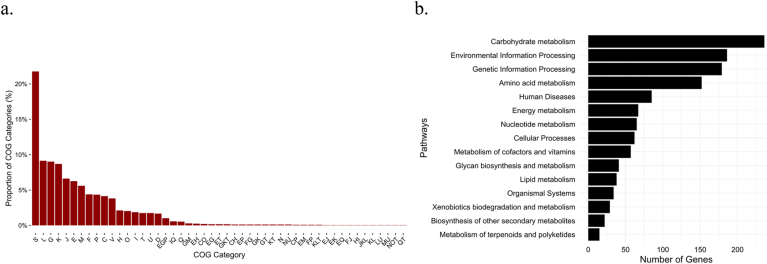
Functional enrichment of genes in *L. paracasei* E. (a) The number of genes assigned in cluster of orthologous group (COG) categories. (b) Genes assigned in KEGG mapper. S - function unknown, L - replication, recombination and repair, G - carbohydrate transport and metabolism, K – Transcription, J - translation, ribosomal structure and biogenesis, E − amino acid transport and metabolism, M - cell wall/membrane/envelope biogenesis, F- nucleotide transport and metabolism, P - inorganic ion transport and metabolism, C - energy production and conversion, V - defense mechanisms, H - coenzyme transport and metabolism, O – post-translational modification protein turnover, chaperones, I- lipid transport and metabolism, T - signal transduction mechanisms, U - intracellular trafficking, secretion, and vesicular transport, D - cell cycle control, cell division, chromosome partitioning, N - cell motility, Q - secondary metabolites biosynthesis, transport and catabolism.

The functional characteristics of the genome was annotated by eggNOG-mapper. Of the 3065 CDSs, 2463 genes (80 %) were assigned to COG categories. As shown in the chart, 22 % of its protein sequences were assigned to S “Function Unknown” category. Followed by L “Replication, recombination and repair” accounting 9.1 % and 9.0 % accounts for “Carbohydrate transport and metabolism” (Fig. 1a). These findings are consistent with previous studies, which also reported a high proportion of proteins in this species being functionally unknown (Kiousi et al., 2022), suggesting that the species remains understudied at the molecular and functional levels. To gain further insight into the metabolic and biological pathways associated with the strain, KEGG Ids extracted from the BlastKOALA were searched against the KEGG Mapper. In total, 1477 KEGG entries were assigned into 199 pathways. There are 236 genes (16 %) engaged in carbohydate metabolism pathway, and 179 genes (12 %) involved in Genetic Information Process pathways (Fig. 1b). The notably large proportion of genes allocated to replication, recombination and repair may represent a genomic signature of this strain's nomadic ecology. However, this observation should be interpreted with caution, as transposable and insertion elements are also classified into this category. The abundance of these mobile elements is, in itself, a common feature in *L. paracasei* strains.

The strain carries eight plasmids. The identification of these plasmids was based on the presence of characteristic plasmid genes, such as plasmid replication and plasmid mobilisation genes. These plasmids exhibit sequence homology to plasmids from other lactic acid bacteria, primarily those of dairy origin, including *L. rhamnosus, Pediococcus parvulus, Lacticaseibacillus zeae, and Loigolactobacillus rennini.* Notably, the smallest plasmid (p08, 4307 bp) of the strain E shares 98 % sequence identity with plasmids from *L. rennini, Levilactobacillus brevis*, and *Lactiplantibacillus plantarum.* The high plasmid homology to other LAB strongly suggests that *L. paracasei* E has been a recipient of horizontal gene transfer (HGT) events, with conjugation being the most probable transfer mechanism.

Lysogeny is a common phenomenon in bacteria of the genus *Lacticaseibacillus* (Jarocki et al., 2023). Searching prophage regions with PHASTEST online tool revealed three intact prophages with sizes 23, 37, 48 kb in the chromosomal contig (Supplementary Table S1). Pei et al. (2021) conducted a comprehensive study on prophages in various lactobacilli species and found that *L. paracasei* has a significantly higher number of intact prophages compared to other *Lacticaseibacillus* species. The majority of *L. paracasei* strains possess up to two intact prophage sequences. Similar findings were found by Jarocki et al. (2023). The CRISPR-Cas system is well known for defending against bacteriophages and other mobile genetic elements (Shabbir et al., 2019). One CRISPR-Cas Type IIA with 21 spacers in *L. paracasei* E was identified (Supplementary Table S2). Even though the array was predicted to be at the evidence level 4, i.e. likely to be complete and functional, it obviously failed to prevent the presence of three intact prophages in the chromosome of the *L. paracasei* E. This is not surprising, as according to Pei et al. (2021), the presence of type II CRISPR-Cas systems does not effectively inhibit the integration of lysogenic phages in lactobacilli.

The evaluation of safety is fundamental to probiotic studies. The antimicrobial potential of *L. paracasei* E was studied *in silico* and *in vivo*. Several bacteriocin genes were identified by using the NCBI database (Supplementary Table S3). In addition, the online bacteriocin finder Bagel 4 was used to predict bacteriocin clusters. Two areas of interests, with several putative bacteriocin genes were found, including genes for bacteriocins garvieacin Q, carnocin_CP52, enterocin_X_chain_beta, and LSEI_2386. The antimicrobial activity was tested by conventional spot-on-lawn method against Gram-positive strains including *B. cereus* ATCC10987, LGG, *L. plantarum* NC8, *Lactococcus lactis* IL1403, *L. monocytogenes* EGD-e, and *S. aureus* ATCC12600. None of the indicators were inhibited by *L. paracasei* E.

*Lactobacilli* are characterized with their large numbers of mobile genetic elements enabling them to exchange genetic information frequently between strains (van Reenen and Dicks, 2011). Based on the genome annotation, there are 63 transposase genes in *L. paracasei* E, which might allow gene recombination and horizontal gene transfer. Thus, it is pivotal to inspect whether the strain possesses any virulence factors or antibiotic resistance genes. For the antibiotic resistance genes, only one strict hit was identified against CARD (Comprehensive Antibiotic Resistance Database) 4.0.0 database: *qacJ*, a part of the small multi-drug resistance (SMR) family, associated with resistance to disinfectants and antiseptics. Regarding possible virulence factors, the online tool VirulenceFinder 2.0 was used. No virulence genes from *L. paracasei* E genome were found. Based on the results from BlastKOALA tool, 3 drug resistance pathways were identified, including beta-lactam resistance and vancomycin resistance and cationic antimicrobial peptide (CAMP) resistance. Among these, only vancomycin resistance has been phenotypically confirmed, while the others require further experimental validation. *In vitro* antibiotic resistance results revealed that *L. paracasei* E exhibited resistance to vancomycin, while being susceptible to a broad spectrum of antibiotics (Supplementary Table S4). In fact, previous research has reported that 79 % of *Lactobacillus* isolates from probiotic products demonstrated vancomycin resistance (Anisimova et al., 2022). Therefore, the vancomycin resistance observed in *L. paracasei* E is not unexpected.

### 3.2 Pan-genome analysis

**Fig. 2:**
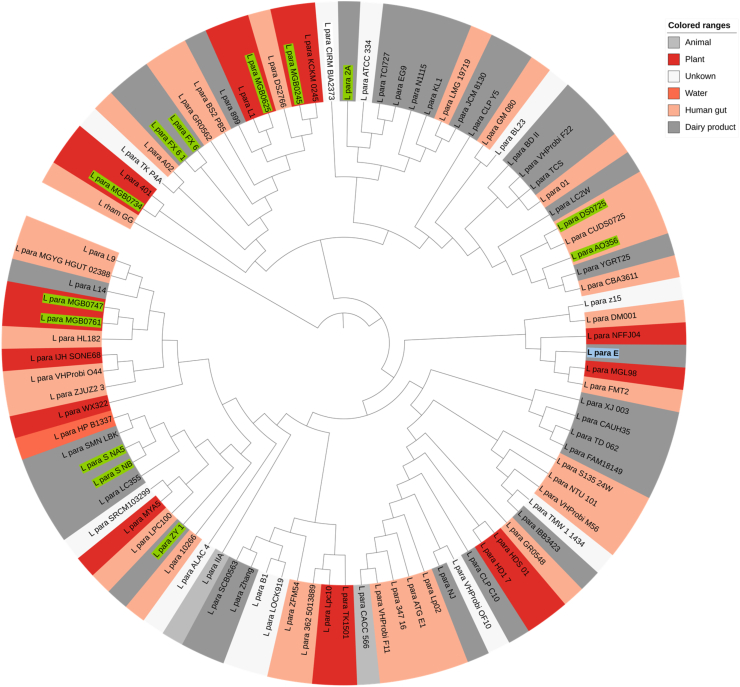
The phylogenetic tree of *L. paracasei* species. *L. paracasei* is abbreviated as L. para. *L. paracasei* strain E highlighted in blue background color. *L. paracasei* subsp. *tolerans* highlighted in green background color.

For pan-genome analysis, 92 complete genomes of *L. paracasei* were downloaded from NCBI. In combined with *L. paracasei* E and an outgroup LGG, we constructed a phylogenetic tree based on single-copy core genes of these genomes (Fig. 2). The results showed that *L. paracasei* E clustered to strains from different isolation sources, including *Homo sapiens* and fermented foods. This suggests that these strains do not form phylogenetic clusters based on their origin, highlighting their ability to thrive in multiple environments. Similar results were reported on *L. paracasei* SP5 in the study by Kiousi et al. (2022). Interestingly, previous research on lactobacilli phylogeny concluded that phylogenetic groups align with metabolic properties (Zheng et al., 2015). No distinct L. *paracasei* subspecies clustering was observed, consistent with the findings of Torres-Miranda et al. (2022), who reported similar results based on ANI, core-genome orthologous protein alignment, and glycoside hydrolase (GH) profile analyses. In addition, the Average Nucleotide Identity (ANI) of all 93 strains was calculated, revealing that the strain E was unique. *L. paracasei* FMT2 was the closest relative from the genomic nucleotide level with an ANI of 89 % similarity. Other closely related strains MGL98 and NFFJ04 with an ANI higher than 85 % have all been used in food production including rice wine and animal feed (Supplementary Table S5).

Based on the pangenome analysis with 92 *L. paracasei* strains*, L. paracasei* E carries 57 unique genes. The list of genes is presented in the Supplementary Table S6. Out of all the 57 unique genes, 34 genes are assigned to functions based on BLAST results. These unique genes are mainly involved in metabolism, signalling and cellular processes. Of particular interest is the identification of a two-component system, which plays a regulatory role in cell adaptation (Serizawa and Sekiguchi, 2005).

### 3.3 Survival test

**Fig. 3:**
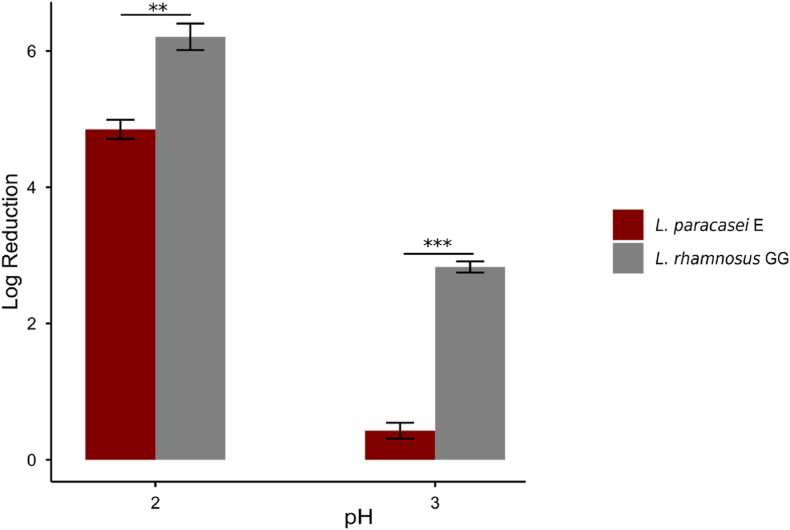
The survival of *L. paracasei* E and LGG in simulated gastric and intestinal fluids. The viable cell counts are expressed as log cfu ml^−1^(mean ± SD). Significant differences within test groups were analyzed by Two-Way ANOVA, and post-hoc pairwise t-tests with Bonferroni corrections (∗∗*p* < 0.01, ∗∗∗*p* < 0.001). Results represent the means of triplicate experiments.

It is essential for probiotic strains to be alive after passing gastrointestinal tract to render their full probiotic health potential. Therefore, resistance to gastric and bile acids and digestive enzymes is crucial (Shimizu et al., 2023), as these components challenge the integrity of the cell membrane, intracellular pH and functional enzymes of the cells (Rodrigues et al., 2019). We assessed the survival of *L. paracasei* E in simulated gastrointestinal conditions, as a comparison with LGG, the probiotic strain known to survive well in intestinal tract. As shown in Fig. 3, only 1-log reduction in cell survival occurred, with nearly half of the *L. paracasei* E cells remaining viable at pH 3. In contrast, LGG exhibited a 3-log reduction in viable cell count, with a survival rate of approximately 1 %. The resilience of both strains decreased sharply at pH 2, where *L. paracasei* E exhibited a substantial 5-log reduction in survival, comparable to 6-log reduction observed in LGG. Thus, *L. paracasei* E demonstrated significantly greater resistance to simulated gastric and intestinal fluids compared to LGG.

The increased resistance observed in strain E therefore suggests the presence of stress-adaptation mechanisms that may support better gastrointestinal persistence and delivery of viable cells to the host gut. We identified a range of genes that are potentially involved in the high survival rate of *L. paracasei* E in simulated gastrointestinal conditions (Supplementary Table S7). We compared the osmotic and acid tolerance genes profile of *L. paracasei* E with LGG. *L. paracasei* E possesses a set of genes that facilitate the uptake and accumulation of osmoprotectants, such as glycine betaine, which functions as an osmolyte that helps the bacteria survive in high-salt environments. For instance, *L. paracasei* E carries one more copy of the osmoprotectant binding genes, *osmF* and *opuCC*, possibly being one reason for its better performance in harsh environments compared to LGG. In addition, gene ACGRWF_01690, encoding for ABC transporter permease in *L. paracasei* E, was found from only 3 out of 93 *L. paracasei* strains. This gene is part of a conserved gene cluster containing also ACGRWF_01695 and ACGRWF_01700. Together, they are predicted to encode an ABC-type transporter complex, which is likely to be involved in the uptake of osmoprotectants (van der Heide and Poolman, 2000).

### 3.4 Quantification of surface-located SpaC

**Fig. 4:**
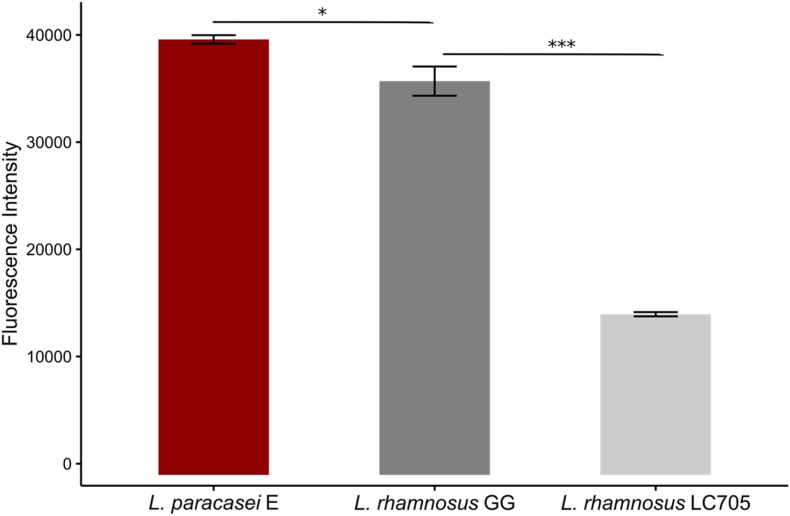
Quantitation of SpaC on the cell surface of LGG, *L. paracasei* E, and *L. rhamnosus* LC705. LGG served as the positive control, while *L. rhamnosus* LC705 was used as the negative control. Statistical significance among groups was determined using one-way ANOVA with post hoc LSD test (∗∗*p* < 0.01, ∗∗∗*p* < 0.001). Data are presented as means ± SD from three independent experiments.

**Fig. 5:**
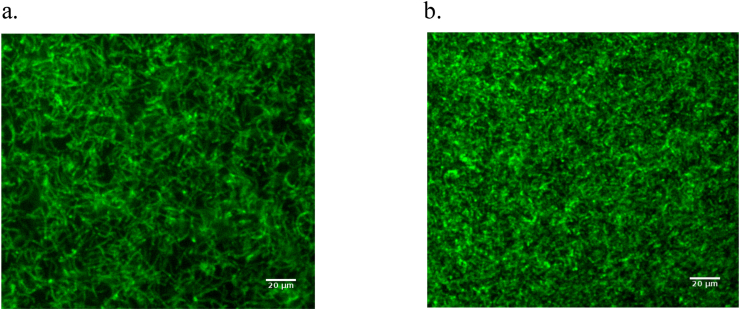
SpaC Immunofluorescent labeling on cell surface of (a) LGG, (b) *L. paracasei* E.

The probiotic effects of LGG are largely attributed to its adhesive heterotrimeric sortase-dependent pili, encoded by the *spaCBA-srtC1* gene cluster (Kankainen et al., 2009). *L. paracasei* E carries two copies of identical LGG-like pilus gene clusters. SpaC pilin has been identified as a key component in bacterial adhesion (Ardita et al., 2014; Segers and Lebeer, 2014). Therefore, we aimed to quantify the SpaC pilins on the cell surface of *L. paracasei* E, compared with LGG, by using In-liquid immunolabeling assay. The specificity of the anti-LGG SpaC antibody toward *L. paracasei* E was supported by 94 % amino acid sequence identity and conserved SpaC protein structure predicted by AlphaFold3 (Supplementary Fig. 2). As shown by the results, more than 2-fold higher fluorescence intensity was detected for LGG and *L. paracasei* E than the pilus-negative strain *L. rhamnosus* LC705. *L. paracasei* E expressed significantly more SpaC compared to LGG (Fig. 4). The SpaC pilins were visualized also by fluorescence microscopy (Fig. 5). Two copies of *spaCBA-srtC1* gene clusters in the strain E may account for higher gene expression level resulting in more pilus proteins, seen as stronger fluorescence. Previously, *L. paracasei* strain IBB3423 (Koryszewska-Bagińska et al., 2019) and LOCK 0919 (Aleksandrzak-Piekarczyk et al., 2016) have been reported to harbour two copies of *spaCBA* gene clusters - one located on the chromosome and the other on a plasmid - and exhibit high adhesion properties. Notably, our study is the first to quantify the SpaC pilin on *L. paracasei* with two chromosomal copies of the *spaCBA-srtC1* gene clusters, suggesting a potential association between gene copy number and elevated protein expression.

### 3.5 Cell surface hydrophobicity

In our first attempts to quantify cell surface SpaC pilin by immunolabeling on microplate (Nunc MaxiSorp ELISA, Thermo Fisher Scientific), we noticed a big difference in the binding efficiency of LGG and *L. paracasei* E, the latter binding much stronger onto the microplate wells (Supplementary Fig. 1). This raised a question about the differences in surface properties of the two strains. Therefore, we studied the hydrophobicity properties of LGG and *L. paracasei* E cell surfaces. Bacterial adherence on inert surfaces such as metal and plastic is affected by the surface physicochemical properties such as charge, hydrophobicity, flagella, pili and exopolysaccharides (EPS) (Botes et al., 2008). Previous research on *Lactobacillus casei* demonstrated that heat shock induces changes in cell surface hydrophobicity, which correspondingly reduced the bacteria's ability to adhere to polystyrene surfaces (Haddaji et al., 2015). To evaluate the cell surface hydrophobicity, we used three different solvents: chloroform (an acidic solvent), hexadecane (an apolar solvent), and ethyl acetate (a basic solvent). These solvents interact with different functional groups on the bacterial surface, providing insights into their hydrophobic characteristics and how these properties affect bacterial adhesion (Zeraik and Nitschke, 2012).

**Fig. 6:**
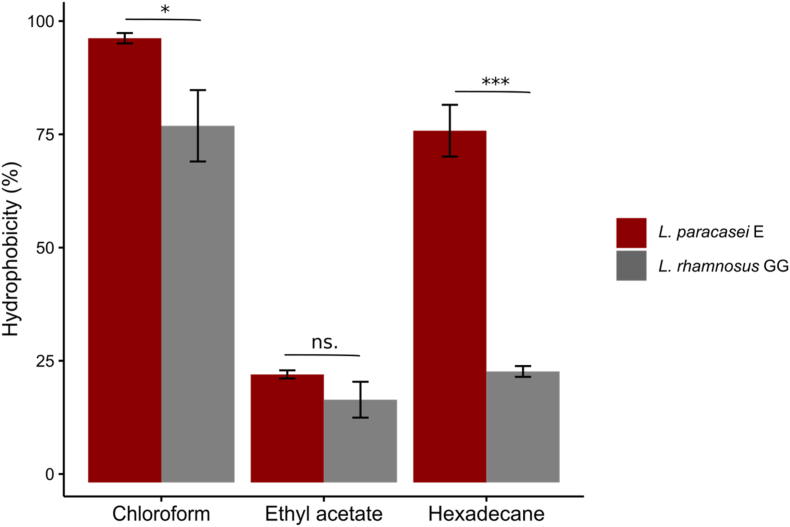
Cell surface hydrophobicity of *L. paracasei* E and LGG to solvents (chloroform, ethyl acetate, hexadecane). Significant differences within test groups were analyzed using an Independent Samples *t*-test (∗*p <* 0.05*, ∗∗∗p <* 0.001*, ns.*: not significant)*.* Results represent the means of triplicate experiments.

As shown in Fig. 6, the higher affinity to chloroform for *L. paracasei* E and LGG over ethyl acetate was an indicative of the predominance of basic properties. Based on Boris et al. (1998) and Chae et al. (2006), strains with over 40 % of hydrophobicity were regarded hydrophobic. The significantly lower affinity to hexadecane for LGG to *L. paracasei* E suggests that *L. paracasei* E cell surface is much more hydrophobic than LGG, which may explain the detachment of cells during the non-ionic detergent wash. Consistently, previous research has also found that the LGG cell surface is hydrophilic (Pithva et al., 2014). Microbial cell surface hydrophobicity is accepted as one of the determinant factors in microbial-host interaction (Deepika and Charalampopoulos, 2010). We analyzed genes related to bacterial cell surface associated proteins in the strain E genome. The gene *licD* caught our attention, since it is absent in LGG, but presents in some of the *L. paracasei* strains including the strain E. The *licD* gene is associated with the modification of cell surface molecules including lipoteichoic acids (LTA) and teichoic acids (TA) (Gisch et al., 2015). LTA and TA play crucial roles in the structural integrity of the bacterial cell wall and in mediating interactions with the host (Shiraishi and Yokota, 2024). Overall, the different hydrophobic profile of these two bacteria indicates differences in surface architecture that may affect initial contact with host tissues and overall adhesion behavior.

### 3.6 Mucus adhesion

**Fig. 7:**
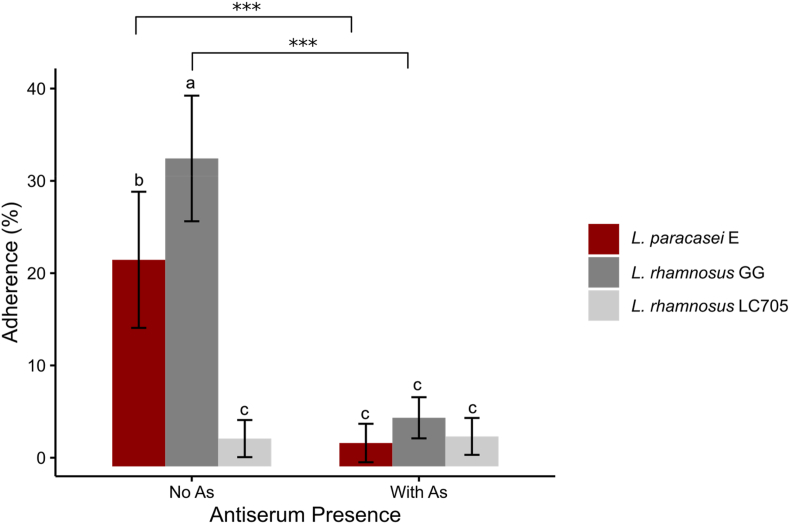
Adherence of LGG, *L. paracasei* E, and the pilusless control *L. rhamnosus* LC705 to porcine mucus in the presence or absence of SpaC antiserum (As). Statistical significance for comparisons among the groups without antiserum was evaluated using one-way ANOVA followed by post hoc LSD testing. Differences between paired observations (with and without SpaC antiserum) were analyzed using paired samples *t*-test (∗∗p < 0.01, ∗∗∗p < 0.001). Bars labeled with different letters indicate statistically significant differences among groups (p < 0.05). Data are presented as means ± SD, based on three independent experiments.

The adhesion of probiotics to the gastrointestinal surface may facilitate their immunological functions and promote the competitive exclusion of pathogens. The mucus layer coating the epithelial cells is where microbiota is directly in contact with its host. We examined the adhesion of *L. paracasei* E to porcine type II intestinal mucus, compared with the highly adherent strain LGG. The adhesion of the poorly adherent negative control strain *L. rhamnosus* LC705 was as low as 3 %, while the adherence of LGG and *L. paracasei* E were 33.36 % and 22.38 %, respectively (Fig. 7). Anti-SpaC antiserum treatment of the cells before interaction with mucus resulted in a significant reduction in the adhesion ability of both LGG and *L. paracasei* E cells. This suggested that the pilus was important in mediating the binding of the cells to mucus. Although L. *paracasei* E exhibited a higher level of SpaC pilin expression and hydrophobic surface, it did not demonstrate stronger adhesion to intestinal surfaces compared to LGG. Bacterial adhesion to intestinal surfaces is a synergistic process influenced by various cell-surface characteristics, including membrane hydrophobicity, the presence of adhesins, and the pili. These factors collectively determine the ability of probiotic bacteria to interact with the host epithelium and mucus layers, a key aspect of probiotic functionality and intestinal colonization (Monteagudo-Mera et al., 2019).

A number of genes potentially associated with cell adhesion were identified (Supplementary Table S8). Previously, it has been reported that *spaCBA-srtC1* gene cluster in LGG play primary role on host-cell attachment (Kankainen et al., 2009). In the strain E, the presence of two copies of the *spaCBA-srtC1* gene cluster may contribute to its relatively high adhesion properties. However, differences were observed in mucus attachment when compared to the LGG. These differences could be attributed to different cell surface hydrophobicity, and the involvement of other proteins in LGG, such as MabA (Vélez et al., 2010) and MBF (von Ossowski et al., 2011). Surface proteins in Gram-positive bacteria are anchored to the cell wall via a universal mechanism involving the LPXTG motif, which causes specific cleavage and anchoring of the N-terminal part of the protein (Navarre and Schneewind, 1994). Upon examination of the LPXTG domain-containing proteins in the genome, we identified a unique gene ACGRWF_07935 present exclusively in the strain E. This distinct genetic feature may suggest that the strain E possesses unique characteristics, potentially contributing to its enhanced adhesive properties compared to other non-adhesive *L. paracasei* strains.

In addition, our results indicated that there were no significant differences in the attachment to human intestinal cell lines for LGG and *L. paracasei* E, though for the strain E, higher adhesion to HT-29 cells (AVE 48–59 %) was observed in two repeats (Supplementary Fig. 2).

## 4 Conclusion

In this study, we expanded characterization of the potential probiotic strain of *Lacticaseibacillus paracasei* strain E through comparative genomic analysis and a series of *in vitro* experiments, including hydrophobicity assays, simulated gastrointestinal survival tests, immunofluorescent labeling of the pilus key component SpaC, and epithelial cell adhesion assays. The strain exhibits potential probiotic attributes comparable to the well-known probiotic strain LGG. Like LGG, *L. paracasei* strain E is free of virulence factors and acquired antimicrobial resistance genes, and it possesses the SpaCBA pilus genes that facilitate adherence to epithelium. Additionally, this strain demonstrates better resistance to simulated gastrointestinal fluids than LGG, and its surface is more hydrophobic than LGG. *L. paracasei* E can be a potential probiotic candidate in food production. Further randomized, controlled clinical trials are warranted to confirm its efficacy in humans.

## CRediT author contribution statement

Nina Zhang: Funding acquisition, Conceptualization, Methodology, Investigation, Data Curation, Formal analysis, Visualization, Writing – Original draft. Antti Karkman: Methodology, Software, Writing – Review & Editing. Kaisa Hiippala: Investigation, Writing – Review & Editing. Reetta Satokari: Resources, Writing – Review & Editing. Timo M. Takala: Conceptualization, Supervision, Writing – Review & Editing. Per E.J. Saris: Conceptualization, Supervision, Resources, Writing – Review & Editing.

## Declaration of generative AI and AI-assisted technologies in the writing process

During the preparation of this work the author(s) used ChatGPT in order to improve the readability and language of the manuscript. After using this tool/service, the author(s) reviewed and edited the content as needed and take(s) full responsibility for the content of the published article.

## Declaration of competing interest

The authors declare that they have no known competing financial interests or personal relationships that could have appeared to influence the work reported in this paper.
