# Supplementary material for: Comparative genomics and characterization of highly adhesive and stress resistant Lacticaseibacillus paracasei strain E
Source: Curr Res Food Sci. 2025 Nov 15;11:101245. doi: 10.1016/j.crfs.2025.101245 (PMC12681685; doi:10.1016/j.crfs.2025.101245)

# Supplementary Materials

## Supplementary Methods

Human colonic epithelial cell lines Caco-2 (ACC169) and HT-29 (ACC299) were purchased from the German Collection of Microorganisms and Cell Cultures (DSMZ, Braunschweig, Germany). Cells were grown at 37°C under an oxic atmosphere with 5% CO_2_. Cell lines were passaged every 3–4 days after reaching 70–80% confluence using TryplExpress (Lonza). HT-29 cells were grown in McCoy 5A medium (Biowest) containing 10% heat-inactivated fetal bovine serum (FBS; Gibco) and 100 U/ml penicillin and streptomycin (PEST, Gibco). Caco-2 cells were cultivated in RPMI 1640 medium (Biowest) supplemented with 20% FBS, nonessential amino acids (1%, NEAA; Lonza), 15 mM HEPES (Lonza), 100 U/ml PEST and 2 mM L-glutamine (Lonza). Passages up to 30 were used in the experiments.

Bacterial adhesion to epithelial cell lines was assessed using a protocol from [Hiippala et al. (2022)](https://helsinkifi-my.sharepoint.com/personal/zhangnin_ad_helsinki_fi/Documents/PAPER1/Lacticaseibacillus_paracasei_E_copy_ref.docx#ref30_hiippala_2022). To radiolabel the bacterial cells, *L. paracasei* E and LGG were cultured in MRS medium supplemented with 10 μl/ml of 17 Ci/mmol [6-^3^H] thymidine (PerkinElmer Scientific). Six technical replicates were used in each experiment. 12.500 Caco-2 or HT-29 cells per well were seeded onto 96-well microplates. [^3^H]Thymidine-labeled bacterial cells were washed three times and adjusted to OD600nm 0.25. After one hour of incubation on the epithelial cell monolayer at 37°C, the bacterial suspensions were removed, and the wells were washed three times to remove the non-adherent bacteria. The adhered bacteria were lysed with 1% SDS-0.1 M NaOH solution overnight at 37°C. Radioactivity was measured with a liquid scintillator (Wallac Winspectral 1414, (PerkinElmer Scientific). The adhesion percentage was calculated relative to the radioactivity of the bacterial suspension initially added to the wells.

## Supplementary Figures

Supplementary Fig. 1. Cell attachment on polystyrene surface. (a) *L.rhamnosus* GG, (b) *L.paracasei* E. With the same amount of cells seeded on the microplate, after washing the wells with PBST, the number of bound LGG cells was considerably lower than *L. paracasei* E cells.

a. b.


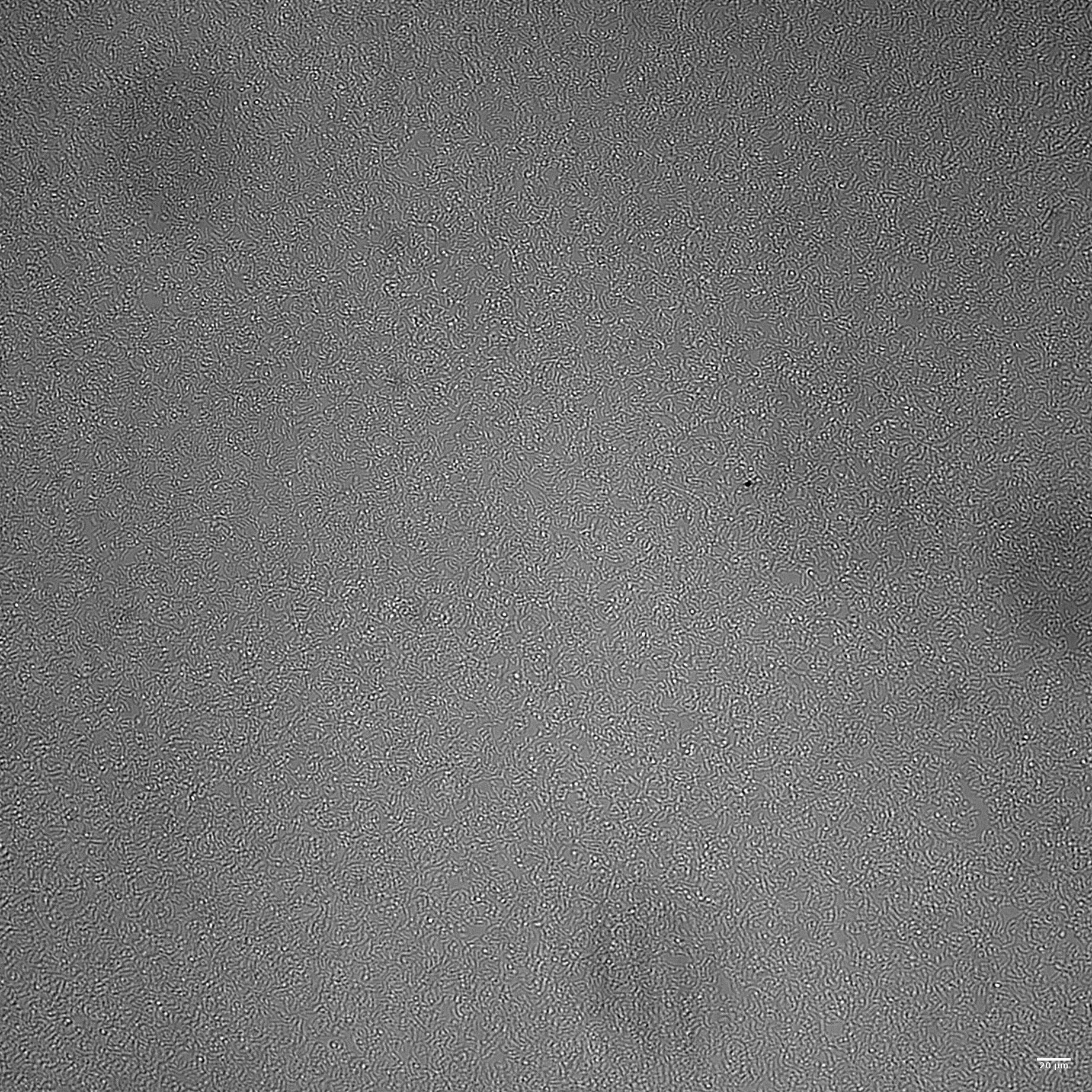

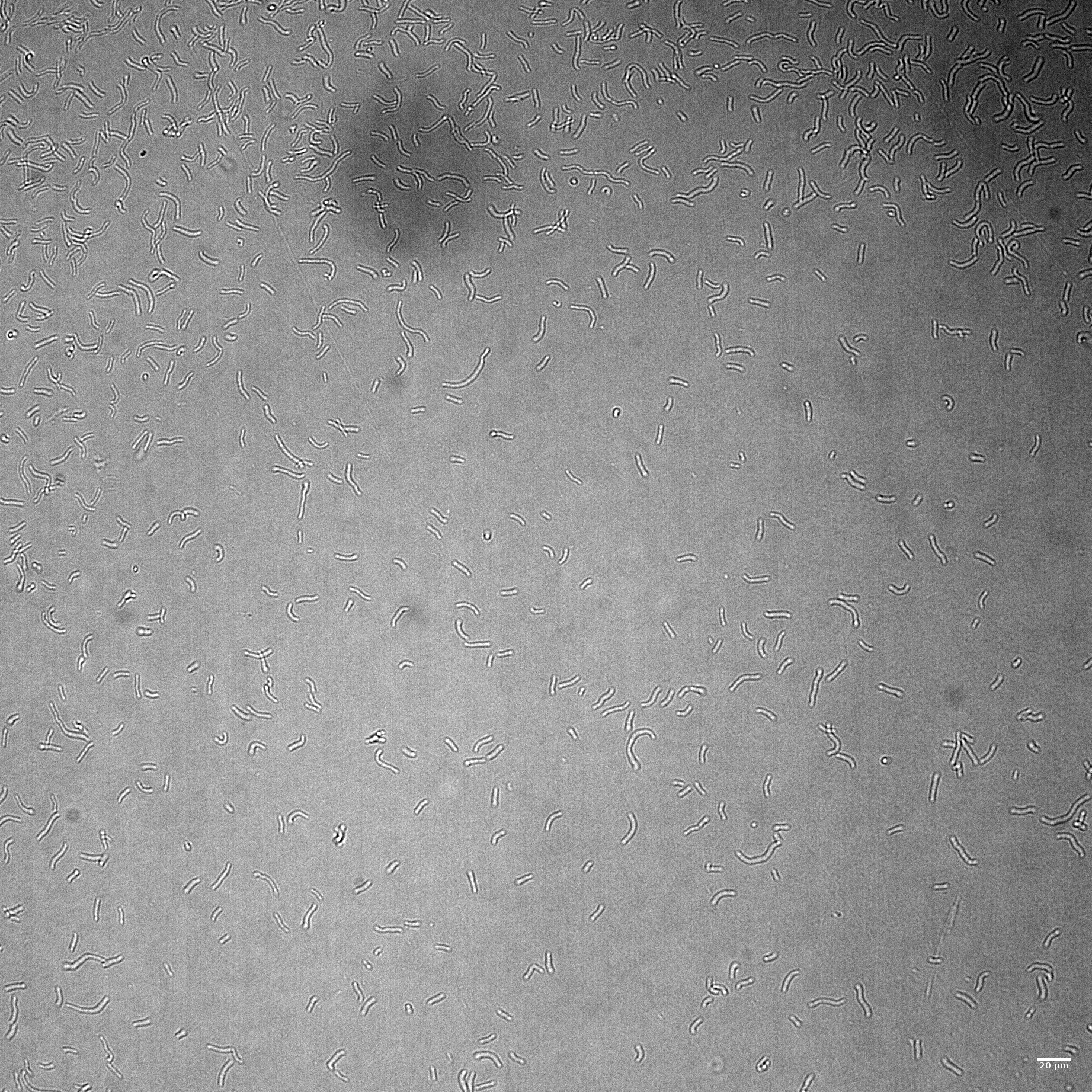


Supplementary Fig. 2. The predicted protein structure of SpaC. (a) *L. rhamnosus* GG, (b) and *L. paracasei* E. With LGG ipTM = -pTM = 0.79; *L. paracasei* E ipTM = -pTM = 0.8.

a.


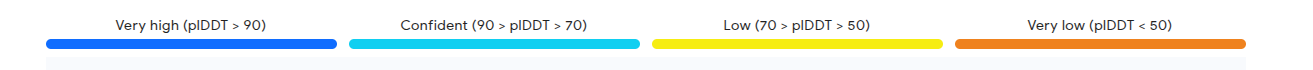


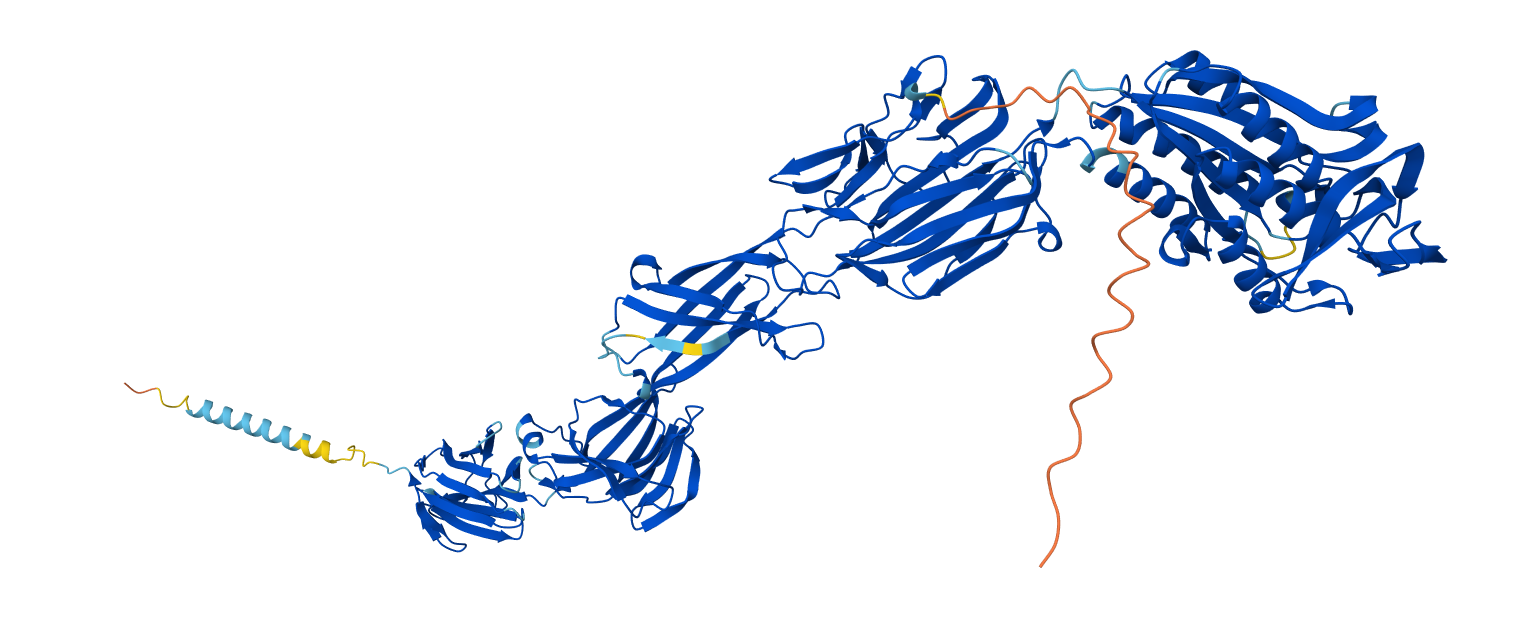


b.


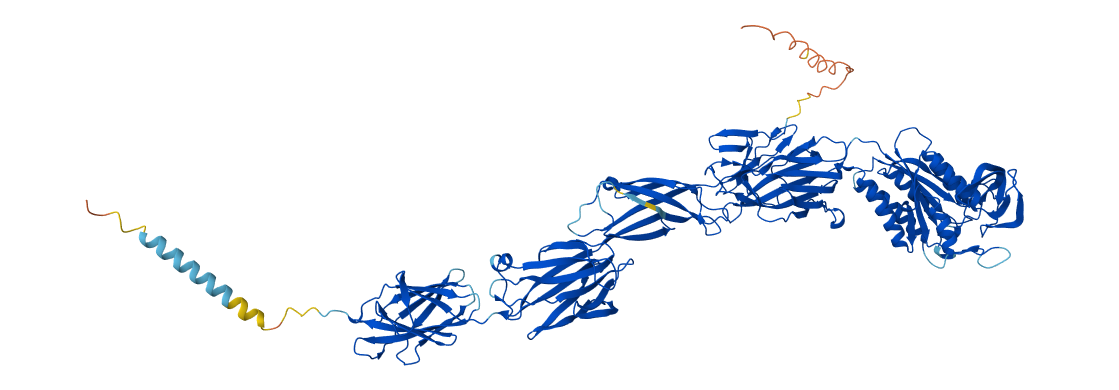


Supplementary Fig. 3. Adhesion of *L. rhamnosus* GG and *L. paracasei* E to human intestinal cell line Caco-2 and HT-29. Significant differences within test groups were analyzed using an Independent Samples t-test (****p <* 0.001*, ns.*: not significant)*.* Results represent the means of triplicate experiments.


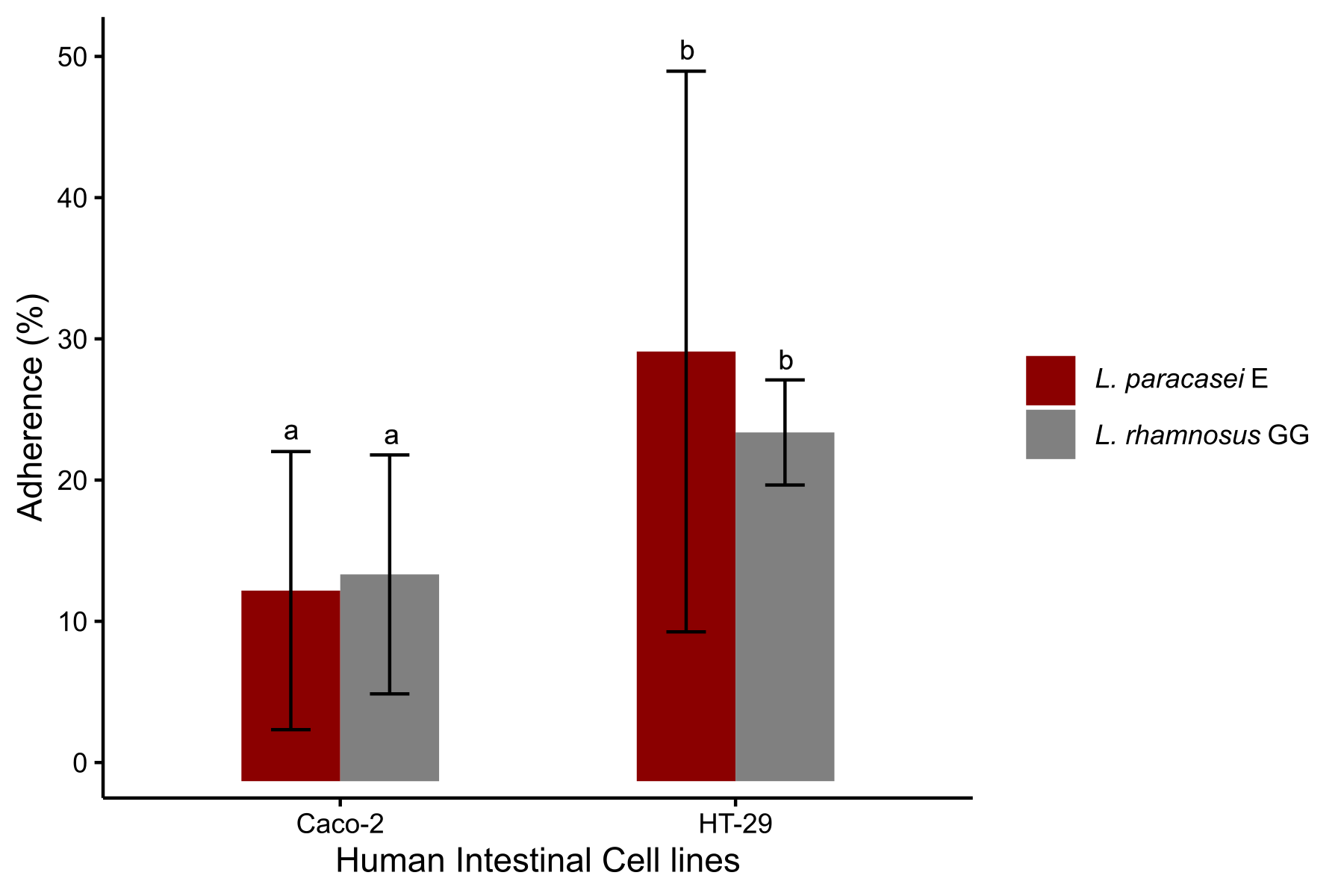

Supplement: Multimedia component 1 [file mmc1.docx]
